# Letter from Philadelphia

**Published:** 1862-04

**Authors:** 


### Correspondence.

Messrs. Editors:—After reading the editorial of "W." in your March number, my first impulse was to let the discussion between us rest there, as he speaks so fair, *comparatively;* but the contrast between what I consider his attack in the January number and his explanation in the March number is so glaring, that I can not forbear brief quotations from both.

In explanation I would say, that it has occurred to me that my manner of speaking of the dental school in this city may have appeared invidious to the other schools, and thereby induced the inference that I claimed *superior* ability for the Faculty here, and such an impression may have provoked the remarks of "W."; but I here disclaim *emphatically* any such thought or intention. What I meant to convey was, that the peculiarly favorable locality of the school—in being in this city—ability to teach on the part of the Faculty, and the character and extent of the class annually, combined to place it in a leading position.

*Extract from Editorial of "W." in January No. "Register."*

"But if 'O. U. C.' means that the 'ability' of the Philadelphia Faculty warrants such a position, we take issue. Like our correspondent, we are outside of all Faculties; and from *personal* acquaintance, we frankly give our opinion, that in all the dental colleges of America, NO FACULTY HAS LESS ABILITY THAN THAT OF THE LEADING SCHOOL, and this statement can not be interpreted into even an apparent disrespect to the Faculty referred to; for *no set of men in existence are more* CONTENTEDLY *conscious of the truth of the statement than the members of this Faculty.*"

*Extract from Editorial of "W." in March No. of "Register."*

"But let us analyze and compare: Our correspondent alluded to the Faculty of the Pennsylvania College. We did, too. Was that 'unwarranted?' *He spoke rather favorably of the Faculty.* WE DID, TOO. Was that '*unmerited?*' *He intimated that the Faculty was able.* WE COULDN'T HELP BUT COINCIDE. Was that a display of 'ill feeling?' We went farther, and intimated that, with all their talents, they were not superior to" (He here introduces the names of the Faculties of the Cincinnati and Baltimore schools, and asks) "is that 'positive insult?'"

Now, I did not mean to claim that the Faculty here were superior to the gentlemen alluded to in the above, nor does my language warrant any such inference, consequently, the allusion to them is uncalled for, and has nothing to do with the question at issue.

The reader will please observe the italicised sentences (the italics are mainly my own), and compare them ; and then point out, if possible, the *harmony !* existing between the two. No, no. If language means anything, the sentiment expressed or meant to be conveyed in the editorial of which the first extract is a part, may be paraphrased thus: "The Faculty of the Pennsylvania College has less ability than that of any other school in America, and they not only know it, but are perfectly contented in that knowledge." Or, more bluntly, "that they are the most ignorant, and happy in being the most ignorant."

This is the point—the gist of the whole matter,—and any explanations, such as that in the March number, no matter how specious or witty, or however well calculated to shift the issue or blind the eyes of the reader, will not satisfy the record.

Here I close, having said four times as much as was intended, and I hope all I need say in this gratuitous, and, to me, unpleasant discussion.

*Remuneration.*—After a somewhat lengthy and extended acquaintaace with the profession, I have become convinced that as a general thing, those who inveigh against what they sneeringly term a mercenary spirit—the pursuit of filthy lucre, etc., in those who speak of fees or remuneration, are the most exorbitant in their charges and the most determined in their collections. To hear them at our conventions, etc., you would suppose they considered it a great privilege to labor for the relief of humanity, and were perfectly willing to leave remuneration to the generosity of the public—were willing to trust, like Elisha, to the ravens ; but when you come to inquire into their practices at home, you discover that they do not practice what they preach, and lawyers, not ravens, are the agents they employ. It is perfectly safe to interpret such utterances as we do dreams, "by contraries."

*Vulcanite* has its disadvantages, as well as advantages. It has so cheapened mechanical dentistry, especially through the Eastern States, as to have almost reduced the profession to a mere trade. Again, it is shamefully abused in being employed indiscriminately, thus damaging the profession financially, and, what is worse, in its reputation.

*The American Dental Convention*, at their last meeting, received a communication on the subject of a World's Convention at London, and an invitation to attend the same, from the "College of Dentists" of London, and which was read and replied to by the Convention, and a resolution passed, "that the officers of the American Dental Convention extend a cordial invitation to the dentists throughout the United States, to attend the World's Dental Convention, to be held in London, in 1862." Yet our Convention adjourned to meet at Trenton Falls, N. Y., on the *second** Tuesday in August, 1862.

For years I have been favorable to a "World's Dental Convention." That our Association, by concert with the Association of London and the dentists of the Continent of Europe, should have a grand Union Convention, an *international*, instead of national Convention for that year. But the time has gone by. The fraternal feeling formerly existing, and which it was desirable to deepen by more immediate ontact and intercourse, has, in consequence of the conduct of the rulers and the press of England during our national troubles, given way to a feeling of indifference, if not dislike, and which feeling may not die, even with this generation. I conclude, therefore, that there will be no representatives from America to the proposed London Convention.

Yours, O. U. C.

*Philadelphia, March* 28, 1862.

---

* Is this right? Heretofore it has been the *first* Tuesday in August.

